# Inhibitory Effect of Select Nitrocompounds and Chlorate against *Yersinia ruckeri* and *Yersinia aleksiciae* In Vitro

**DOI:** 10.3390/pathogens11111381

**Published:** 2022-11-19

**Authors:** Elizabeth A. Latham, Robin C. Anderson, Lauren R. Wottlin, Toni L. Poole, Tawni L. Crippen, Wayne D. Schlosser, Roger B. Harvey, Michael E. Hume

**Affiliations:** 1 Department of Animal Science, Texas A&M University, College Station, TX 77843, USA; 2 Southern Plains Agricultural Research Center, Food and Feed Safety Research Unit, United States Department of Agriculture/Agricultural Research Service, College Station, TX 77845, USA; 3 United States Department of Agriculture/Food Safety Inspection Service, Southern Plains Area Research Center, College Station, TX 77845, USA

**Keywords:** antimicrobial, chlorate, nitrocompound, *Yersinia ruckeri*, *Yersinia aleksiciae*

## Abstract

*Yersinia ruckeri* is an important fish pathogen causing enteric redmouth disease. Antibiotics have traditionally been used to control this pathogen, but concerns of antibiotic resistance have created a need for alternative interventions. Presently, chlorate and certain nitrocompounds were tested against *Y. ruckeri* as well as a related species within the genus, *Y. aleksiciae*, to assess the effects of these inhibitors. The results reveal that 9 mM chlorate had no inhibitory effect against *Y. ruckeri*, but inhibited growth rates and maximum optical densities of *Y. aleksciciae* by 20–25% from those of untreated controls (0.46 h^−1^ and 0.29 maximum optical density, respectively). The results further reveal that 2-nitropropanol and 2-nitroethanol (9 mM) eliminated the growth of both *Y. ruckeri* and *Y. aleksiciae* during anaerobic or aerobic culture. Nitroethane, ethyl nitroacetate and ethyl-2-nitropropionate (9 mM) were less inhibitory when tested similarly. Results from a mixed culture of *Y. ruckeri* with fish tank microbes and of *Y. aleksiciae* with porcine fecal microbes reveal that the anti-*Yersinia* activity of the tested nitrocompounds was bactericidal, with 2-nitropropanol and 2-nitroethanol being more potent than the other tested nitrocompounds. The anti-*Yersinia* activity observed with these tested compounds warrants further study to elucidate the mechanisms of action and strategies for their practical application.

## 1. Introduction

*Yersinia ruckeri* is a fish pathogen that causes an economically important disease among fish. The disease, called enteric redmouth disease, mainly affects salmonids (trout and salmon) although its emergence as an important pathogen causing infections and disease outbreaks in numerous fish species outside the Salmonidae family has been reviewed [1]. Disease transmission occurs via the spread of the bacteria from infected fish, birds or other animals [2]. Infection occurs predominantly via bacterial attachment and invasion of tissues within the gills, although infections via attachment and invasion of the epithelial cells of the gastrointestinal tract or skin along the lateral line have also been reported [2,3]. Experimental evidence indicates that virulent serotypes of *Y. ruckeri* may sequester and remain viable within macrophages of carrier animals for up to 2 weeks, and can survive within fresh or moderately salty aquatic habitats for up to 4 months after an enteric redmouth disease outbreak [4,5].

Current methods for the prevention or treatment of enteric redmouth disease caused by *Y. ruckeri* involve the administration of vaccines, antibiotics or other antimicrobials [1,6,7]. While these interventions have been efficacious in the past, concern exists that their continuous use may lead to the emergence of *Y. ruckeri* strains exhibiting decreased immunologic response to vaccines or, in the case of repeated antibiotic use, may ultimately contribute to the emergence and dissemination of antibiotic resistance in the aquatic environment at large [1,6,7,8,9,10,11,12]. Accordingly, the aquaculture industry is interested in finding new technologies to prevent and treat enteric redmouth disease.

Like other members of the family Enterobacteriaceae, *Yersinia* are facultatively anaerobic and able to reduce nitrate to nitrite. Some members of the Enterobacteriaceae, such as *Escherichia coli* and *Salmonella*, can be killed by chlorate, which may be co-metabolized by membrane-bound nitrate reductases to yield cytotoxic chlorite [13]. Additionally, results from earlier studies demonstrated that the bactericidal effect of chlorate against *E. coli* and *Salmonella* was markedly enhanced by co-treatment with certain short-chain nitrocompounds or by the nitrocompounds alone, the latter exhibiting antimicrobial activity at concentrations less than 0.20% vol/vol. [13]. However, the mechanism of the nitrocompound-caused antimicrobial activity was not clearly defined [13]. The objective of this study was to investigate the antimicrobial effects of chlorate and certain short-chain nitrocompounds on the growth of *Y. ruckeri*. Recognizing that *Y. ruckeri* is the most distantly related of all the species within the genus, another more centrally related non-pathogenic member of the genus, *Yersinia aleksiciae*, was also tested for comparison.

## 2. Materials and Methods

### 2.1. Tests with Pure Cultures

*Yersinia ruckeri* (YRNC10 Serovar 1, biotype 2, North Carolina 2003) was provided by Dr. David Pasnik, USDA/ARS, Aquatic Animal Health Research Unit in Auburn, AL, USA. *Yersinia aleksiciae* strain 313 was a stock culture preserved in 20% glycerol at −80 °C at the USDA, Food and Feed Research Unit (College Station, TX, USA). Initially ascribed presumptively via classical biochemical identification tests as *Yersinia enterocolitica*, the identification of this bacterium was confirmed as *Y. aleksiciae* based on whole genome sequence analysis by the USDA, National Veterinary Services Laboratory (Ames, Iowa, USA). Annotation of the *Y. aleksiciae* whole genome sequence (deposited to NCBI BioProject under accession number PRJNA854595) was accomplished using RAST with default parameters [14]. For routine maintenance and pure culture tests, both species were grown anaerobically or aerobically, as indicated, in half-strength BBL^TM^ Brain Heart Infusion (BHI) broth (Becton Dickinson and Company, Sparks, MD, USA). The half-strength medium, prepared as per manufacturer’s instructions, except using half the recommended amount of powder ingredient, was used to avoid confounding effects of excessive acid production during fermentative growth. The medium was distributed to 13 × 100 mm glass culture tubes prior to steam sterilization in an autoclave, which were then aseptically closed with previously sterilized screw caps. For anaerobic medium, sterile broth was equilibrated overnight in a 90 % N_2_: 5 % CO_2_: 5 % H_2_ atmosphere in a Bactron Anaerobic Chamber (Sheldon Manufacturing, Inc., Cornelius, OR, USA) that was aseptically closed with presterilized Hungate screw caps with fitted stoppers (Bellco Glass, Vineland, NJ, USA). Treatments were administered prior to inoculation via the addition of small volumes (<0.5 mL) of concentrated stock solutions. The doses used in this study were based on results reported earlier [15]. *Yersinia ruckeri* cultures were incubated at 28 °C and *Y. aleksiciae* cultures were incubated at 37 °C, unless indicated otherwise, and growth was measured as changes in optical density (OD) at 600 nm using a spectrophotometer (Spectronic 20D, Thermo Spectronic Inc., Madison, WI, USA) at 600 nm. Mean specific growth rates were calculated during the steepest part of logarithmic growth as (ln OD time 2 − ln OD time 1)/(time 2 − time 1) [16].

### 2.2. Tests with Mixed Microbial Populations

To test the effects of select nitrocompounds against *Y. ruckeri* among mixed populations in fish water, freshly collected fish tank water was collected from a continuously well-water-supplied 38-liter culture tank rearing juvenile Nile tilapia, *Oreochromis niloticus*, at the Texas A&M Aquacultural Research and Teaching Facility (College Station, TX, USA). The temperature of the fish tank water at the time of collection was 24.4 °C and dissolved oxygen concentration was 6.33%. The collected fish water was returned to the laboratory and distributed (10 mL/tube) under ambient atmosphere conditions and within 30 min of collection to 18 × 150 mm culture tubes preloaded with 0.3 mL of water or 300 mM stock solutions of respective nitrocompounds to achieve 9 mM treatment. The tubes were immediately closed with rubber stoppers upon receiving the fish water preparation and incubated aerobically at 28 °C. Tests of effects of select nitrocompounds against *Y. aleksiciae* in mixed populations of porcine fecal microbes, arbitrarily selected as an animal source, were accomplished using freshly voided feces collected from a mature, non-pregnant, non-lactating sow at the Texas A&M University, College of Veterinary Medicine (College Station, TX, USA). The collected feces were returned to the lab within 30 min of collection where 0.8 g was mixed with 400 mL of half-strength BHI broth flushed continuously for 30 min prior to and during mixing with 100% N_2_ gas and then inoculated with 0.8 mL of an *Y. aleksiciae* culture grown overnight in half-strength BHI. After 20 min of mixing, 10 mL of the fecal/*Y. aleksiciae* suspension was transferred under a continuous flow of N_2_ gas to 18 × 150 mL crimp top tubes preloaded with stock nitrocompound solutions as described above. The tubes were immediately closed with rubber stoppers, crimped and then incubated at 37 °C. For the mixed culture tests with *Y. ruckeri* or *Y. aleksiciae*, untreated control cultures and treated cultures were incubated in triplicate. For these mixed culture tests, strains of *Y. ruckeri* and *Y. aleksiciae* were made resistant to 25 µg/mL novobiocin and 20 µg/mL nalidixic acid via serial transfer in BHI broth with gradually increasing concentrations of both antibiotics and these strains were used to facilitate their recovery from the wildtype microflora. The antibiotics were purchased from Sigma-Aldrich (St. Louis, MO, USA). Fluid samples (1 mL) were collected from the cultures after 0 and 24 h, and immediately serially diluted in 0.4 M sodium phosphate buffer (pH 6.5) and plated to BBL^TM^ MacConkey agar (Becton Dickinson) supplemented with 25 µg/mL novobiocin and 20 µg/mL nalidixic acid for viable plate count enumeration of the challenge strains. Fresh samples of the fish tank water and fecal suspensions that were not inoculated with the challenge *Yersinia* were also plated to non-antibiotic McConkey agar to check for the presence of wildtype *Yersinia*. Colonies exhibiting distinct morphological characteristics of the *Y. ruckeri* or *Y. aleksiciae* strains after 48 h aerobic incubation at 28 °C on the plated the novobiocin- and nalidixic-acid-supplemented medium were counted. For quality control, representative cultures were confirmed as *Y. ruckeri* and *Y. aleksiciae* via comparison of results from 16S rRNA gene sequence analysis, and biochemical and antimicrobial resistance characterization to the original parent strains.

### 2.3. Statistical Analysis

Mean specific growth rates and maximum optical densities at 600 nm determined during pure culture tests were analyzed for effects of treatments and, when applicable, for the effects of atmosphere (anaerobic versus aerobic) or temperature (28 °C versus 37 °C), and the potential treatment by atmosphere or treatment by temperature interactions using a general analysis of variance. Due to differences in the times of recording of optical density measurements within some of the separate experiments, the resultant mean specific growth rates for cultures treated alike between experiments varied. Consequently, to avoid confounding issues due to the interexperimental differences, all statistical tests of effects of the independent variables and potential interactions were analyzed independently within each experiment, and any comparisons made between experiments were considered qualitatively only. Within the sixth experiment, log_10_ transformations of colony forming units/mL determined during the mixed culture studies were compared for treatment effects using a completely randomized analysis of variance. Main effects were considered significant at *p* < 0.05, and mean comparisons were accomplished using an LSD All-Pairwise Comparisons test. All statistical analyses were performed using Statistix 10 Analytical Software (Tallahassee, FL, USA).

## 3. Results

### 3.1. Tests with Pure Cultures

Results from the first experiment comparing the effects of chlorate, nitrate, 2-nitropropanol or combinations of nitrate with chlorate or 2-nitropropanol (each at 9 mM) are shown in Table 1. Chlorate, at 9 mM, did not inhibit the growth of *Y. ruckeri* but did inhibit the growth of *Y. aleksiciae* when cultured anaerobically in half-strength Brain Heart Infusion broth, as mean specific growth rate and maximum optical density of *Y. aleksiciae* were decreased (*p* < 0.05) when compared to nontreated control cultures (Table 1). The susceptibility of *Y. aleksiciae* to chlorate suggests the presence of a functional membrane-bound respiratory nitrate reductase. Indeed, annotation of *Y. aleksiciae*’s whole genome sequence predicted the presence of genes for a putative respiratory nitrate reductase (Table 2). Co-supplementation of both chlorate and nitrate (each at 9 mM) had no effect on growth rates or maximum optical densities of either *Y. ruckeri* or *Y. aleksiciae* when compared to controls (Table 1). Administration of 9 mM 2-nitropropanol decreased (*p* < 0.0001) growth rates and maximum optical densities of *Y. ruckeri* and *Y. aleksiciae* (Table 1).

Results from the second experiment testing the effects of temperature (28 °C versus 37 °C), individual administration of 2-nitropropanol and 2-nitroethanol (each at 9 mM) and the potential interaction between temperature and nitro-treatment are presented in the text below. Accordingly, the main effects of nitro-treatment (*p* = 0.0002; standard error of the mean, SEM = 0.023), temperature (*p* < 0.0001, SEM = 0.019) and a nitro-treatment–temperature interaction (*p* = 0.0277, SEM = 0.032) were observed on mean specific growth rates during anaerobic pure culture of *Y. ruckeri*. Comparisons of least squares means of the interaction revealed that growth rates were highest for untreated cultures grown at 28 °C (0.359 h^−1^), intermediate for 2-nitropropanol- and 2-nitroethanol-treated cultures grown at 28 °C (0.108 and 0.094 h^−1^, respectively) and lowest for control and nitro-treated cultures grown at 37 °C (<0.056 h^−1^). Similarly, main effects of nitro-treatment, temperature and a nitro-treatment–temperature interaction were observed (*p* < 0.0001; SEM = 0.002, 0.001 and 0.002, respectively) on maximum optical densities achieved during pure culture of *Y. ruckeri*. In this case, least square means of the maximum optical density at 600 nm were highest for untreated cultures grown at 28 °C (0.432), intermediate for controls grown at 37 °C (0.041) as well as 2-nitroethanol- and 2-nitropropanol-treated cultures grown at 28 °C (0.035 and 0.028, respectively), and lowest for 2-nitroethanol- and 2-nitropropanol-treated cultures grown at 37 °C (0.014 and 0.012, respectively).

For pure cultures of *Y. aleksiciae* grown similarly, main effects of nitro-treatment (*p* = 0.0001; SEM = 0.025) and of temperature were observed (*p* = 0.0156; SEM = 0.020) on mean specific growth rates, but not of nitro-treatment–temperature interaction (*p* = 0.5569; SEM = 0.035). For the main effect of nitro-treatment, mean specific growth rates were higher for untreated *Y. aleksiciae* cultures than cultures treated with 9 mM 2-nitropropanol or 9 mM 2-nitroethanol (0.335 versus 0.054 and 0.098 h^−1^, respectively). For the main effect of temperature, main effect mean specific growth rates were higher for *Y. aleksiciae* cultures grown at 37 °C than at 28 °C (0.203 versus 0.122 h^−1^, respectively). Analysis of maximum optical densities achieved at 600 nm revealed a nitro-treatment–temperature interaction (*p* = 0.0001; SEM = 0.008) during anaerobic pure culture of *Y. aleksiciae*. In this case, maximum optical densities were highest for untreated control cultures grown at 28 °C (0.465), intermediate for untreated controls grown at 37 °C (0.310), and lowest for 2-nitropropanol- and 2-nitroethanol-treated cultures, which did not achieve maximum optical densities above 0.015 regardless of incubation temperature.

**Figure 1:**
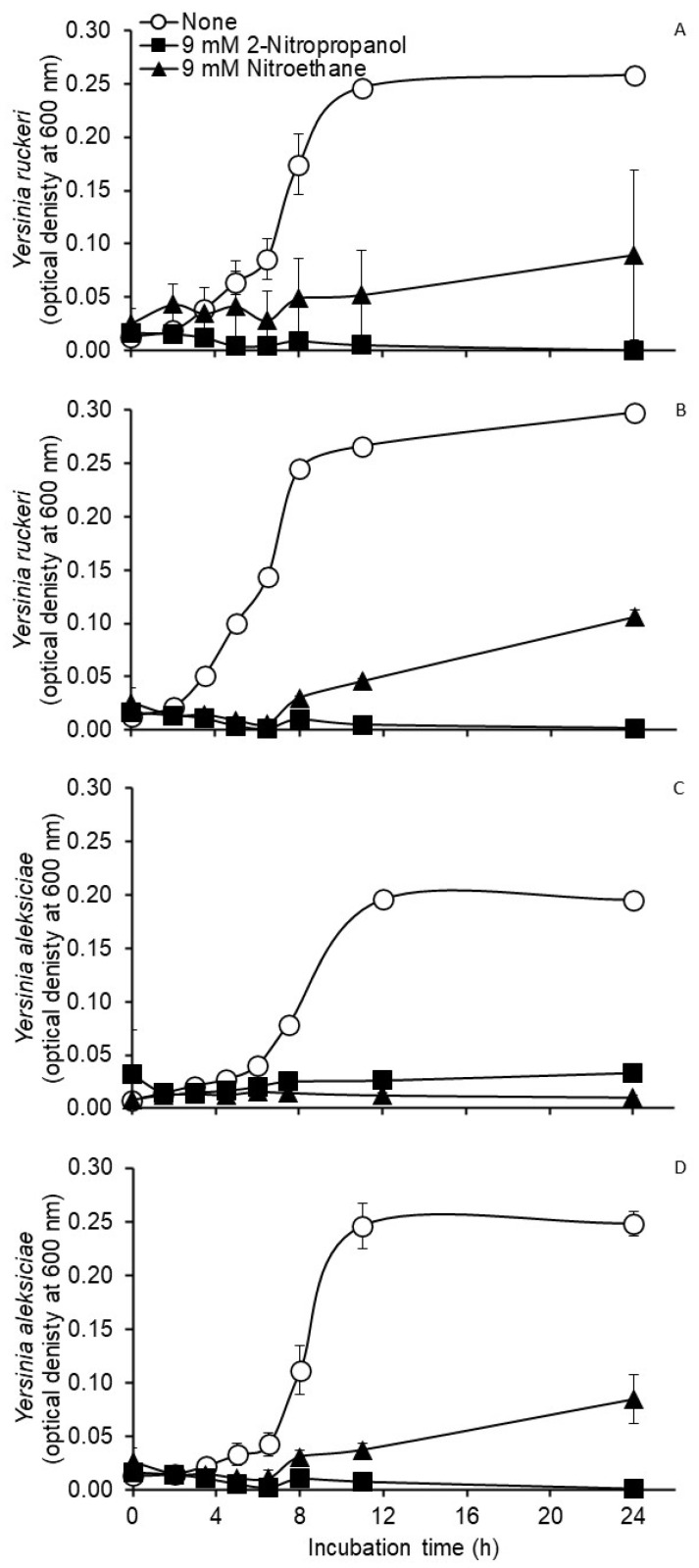
Growth curves of *Yersinia ruckeri* and *Yersinia aleksiciae* during anaerobic (**A** and **B**, respectively) and aerobic (**C** and **D**, respectively) culture in half-strength Brain Heart Infusion broth treated without (open circles) or with 9 mM 2-nitropropanol (closed squares) or 9 mM nitroethane (closed triangles). *Yersinia ruckeri* cultures were incubated at 28 °C and *Y. aleksiciae* cultures were incubated at 37 °C. Values are the mean ± SD from *n* = 3 cultures.

Results from the third experiment with *Y. ruckeri* and *Y. aleksiciae*, designed to compare the effects of aerobic versus anaerobic atmosphere, 2-nitropropanol and a structurally similar compound, nitroethane (each at 9 mM), as well as the possible interaction between atmosphere and nitro-treatment on growth characteristics, are presented in Figure 1. While the main effects of nitro-treatment were observed (*p* < 0.0001) on mean specific growth rates and maximum optical densities of *Y. ruckeri* and of *Y. aleksiciae* when compared to untreated controls, main effects of atmosphere or potential interactions between atmosphere and nitro-treatment were not observed (*p* > 0.05) (Figure 1). The later findings clearly indicate that the inhibitory activity of the nitrocompounds was not impacted during anaerobic or aerobic metabolism.

Results from the fourth experiment testing the comparative anti-*Yersinia* effects of related nitrocompounds and commonly used alcohol disinfectants are presented in Table 3. Nitroethane was less potent as an antimicrobial against both *Y. ruckeri* or *Y. aleksiciae* than the nitro-alcohols, the latter appearing to be near equally effective (Table 3). Ethyl nitroacetate and ethyl-2-nitropropionate exhibited activity more similar to nitroethane than to the nitro-alcohols (Table 3), which suggests that the alcohol functional group contributes, at least partially, to the antimicrobial activity of the nitro-alcohols. At 9 mM, 2-nitropropanol, 3-nitropropanol, 2-nitroethanol, nitroethane, ethyl nitroacetate and ethyl-2-nitropropionate would be equivalent to 0.08, 0.08, 0.06, 0.06, 0.11 and 0.12% (vol/vol), respectively.

Results from a fifth experiment conducted as a dose titration trial reveal that doses as low as 2.25 mM 2-nitropropanol retained significant anti-*Yersinia* activity, as evidenced by decreased (*p* < 0.05) mean specific growth rates when compared to untreated controls (Table 4). However, maximum optical densities achieved by cultures grown with 2.25 mM 2-nitropropanol or nitroethane were not as low as for cultures incubated with 4.5 or 9 mM nitrocompound, thus indicating that optimum potency may be between 2.25 and 4.5 mM for the nitrocompounds tested in this study (Table 4).

### 3.2. Tests with Mixed Cultures

Results from the sixth experiment, measuring the effects of nitrocompound treatment on viable recovery of challenge strains of *Y. ruckeri* and of *Y. aleksiciae* during mixed culture with fish tank and of porcine fecal microbes, are presented in Table 5. When *Y. ruckeri* was cultured aerobically at 28 °C for 24 h with indigenous populations of microbes present within freshly collected tilapia farm tank water, the anti-*Yersinia* activity of 2-nitropropanol and 2-nitroethanol was clearly apparent, with much lesser or no bactericidal activity achieved with nitroethane, ethyl nitroacetate, ethyl 2-nitropropionate or sodium chlorate (Table 5). The 28 °C temperature was used for this mixed culture study to be the equivalent of mid-summer water temperature in Texas. Similar results were achieved when *Y. aleksiciae* was cultured anaerobically with freshly collected indigenous populations of porcine fecal bacteria (0.2% wt/vol) at 37 °C. In this case, the 37 °C temperature was used to simulate the body temperature of an adult pig, which was arbitrarily chosen as the model fecal donor for this study (Table 5).

## 4. Discussion

The observed inhibitory effect of chlorate against *Y. aleksiciae* is unexpected as *Yersinia* are reported to express a periplasmic nitrate reductase rather than a membrane-bound nitrate reductase [17,18]. Unlike the membrane-bound nitrate reductases, periplasmic nitrate reductases generally do not appreciably catalyze the reduction of chlorate to cytotoxic chlorite [17,18]. Evidence from the mixed culture study with porcine fecal suspensions reveals that the anti-*Y. aleksiciae* effect of chlorate addition was bactericidal, as viable colony counts of *Y. aleksiciae* were decreased by approximately 1 log_10_ CFU/mL when compared to initial counts at the start of incubation.

Considering, however, that chlorate by itself is rather innocuous, it seems likely that the anti-*Y. aleksiciae* effect of chlorate addition may be due to chlorite being formed. Annotation of the *Y. aleksiciae* genome made available by the NVSL revealed the presence of genes coding for respiratory nitrate reductase chains that (Table 2), if expressed, may provide an explanation for the enzymatic reduction of chlorate to chlorite in the present study. The NCBI standard protein Blast, BLASTP, of the four putative *Y. aleksiciae* respiratory nitrate reductase chains revealed the closest identity match to be *Y. mollaretii*, a non-human pathogen closely related to and formerly classified as *Y. enterocolitica* [19], and having little or no sequence identity to *E. coli* K-12 or *Y. ruckeri* (Table 2). We cannot confirm from the present results, however, that the genes encoding the respiratory nitrate reductase chains may be translated into a functionally active nitrate reductase able to co-metabolize chlorate to chlorite. Consequently, we cannot exclude the possibility that chlorite, if produced, may be produced via nonspecific reduction mechanisms of enzymes other than respiratory nitrate reductase. For instance, the specificity of the *Y. aleksiciae* periplasmic nitrate reductase may be sufficiently low enough to nonspecifically catalyze the reduction of chlorate to chlorite, or, alternatively, the anti-*Yersinia* effect of chlorate against *Y. aleksiciae* may be due to the nonspecific reduction of chlorate to chlorite by enzymes involved in tetrathionate respiration. The genes for tetrathionate reductase activity (*ttr*) as well as cobalamin synthesis (*cob*) and 1,2-propanediol catabolism (*pdu*) have been reported to be lacking in *Y. ruckeri*, which was unaffected by chlorate treatment, whereas these genes are present in *Y. aleksiciae* and most other, but not all, *Yersinia* [20]. The other *Yersinia* species lacking genes for tetrathionate reductase (*ttr*) are *Y. pseudotuberculosis*, *Y. pestis* and *Y. similis* [17]. According to the above hypothesis, these *ttr*-negative species would be expected to exhibit no inhibitory response due to chlorate, much like that observed with *Y. ruckeri*. In the case of *Y. aleksiciae*, the finding that the inhibitory effect of chlorate was diminished or overcome with co-administration of 9 mM nitrate (Table 1) suggests that nitrate may have downregulated expression of the tetrathionate respiration pathway, as would be expected for the regulated activity [21,22]. Alternatively, nitrate may have been a competing substrate for the operative reductase potentially used by this bacterium [23]. The chlorate-caused inhibition of the mean specific growth rate observed with *Y. aleksiciae* in the present study was modest, with a decrease of about 20% from that of untreated controls. By comparison, the anti-*E. coli* O157:H7 activity of chlorate published earlier caused a decrease in mean specific growth rates of 80% or more during growth with 5 to 10 mM chlorate [24]. Accordingly, is seems reasonable to suspect the potential of chlorate as an anti-*Yersinia* treatment may be impractical.

Several short-chain nitrocompounds, such as 2-nitropropanol, 2-nitroethanol and nitroethane, have been reported to exhibit bactericidal activity against a variety of Gram-negative (*E. coli* O157:H7, *Campylobacter* and *Salmonella* Typhimurium) as well as Gram-positive (*Listeria monocytogenes* and *Staphylococcus aureus*) pathogens [13,15,25,26,27,28,29]. Certain nitrocompounds have also been shown to inhibit the growth of uric-acid-degrading bacteria, as well as rumen methanogens during pure culture, and to inhibit uric acid degradation in poultry litter and rumen-methane-producing activity by mixed populations of rumen microbes in vitro [28,29,30,31,32,33,34,35,36]. The anti-methanogenic activity of the nitrocompounds has also been demonstrated in vivo [37,38,39,40,41]. Additionally, Viedma et al. [42] reported the anti-staphylococcal effect of 2-nitropropanol in baking ingredients. In some cases, chlorate has been reported to act synergistically with certain nitrocompounds [13,15]. Results from the present study demonstrate the anti-*Yersinia* activity of the tested nitrocompounds during pure as well as during mixed culture of *Y. ruckeri* with fish tank microbes and *Y. aleksiciae* with porcine fecal bacteria, with 2-nitropropanol and 2-nitroethanol being more potent than the other tested nitrocompounds. As alluded to earlier, the anti-*Yersinia* activity of the tested nitrocompounds appeared to be bactericidal in nearly all cases, as colony counts during mixed culture were decreased compared to initial numbers, the exceptions being ethyl nitroacetate and ethyl-2-nitropropionate during mixed culture of *Y. ruckeri* with fish tank microbes. In the present study, the combined administration of 2-nitropropanol with chlorate was no more effective in inhibiting the mean specific growth rates and maximum optical densities of *Y. ruckeri* or *Y. aleksiciae* than the administration of 2-nitropropanol alone. Thus, it appears that 2-nitropropanol, being much more potent than chlorate, was the dominant inhibitor. As expected, untreated control cultures of *Y. ruckeri* grew to a much greater degree at 28 °C than at 37 °C, yet, the inhibitory effect of the nitrocompounds was markedly greater during growth at 37 °C than at 28 °C. This latter finding is likely of little practical importance; however, maximum optical densities at 600 nm achieved during the growth of nitro-treated cultures at 28 °C were inhibited by more 90%, when compared to controls grown likewise. Growth of *Y. aleksiciae* in untreated control cultures was also greater at 28 °C than 37 °C, but in this case, the maximum optical density achieved at 28 °C was only 33% greater than that when grown at 37 °C, and maximum optical densities were again decreased > 90% in nitro-treated cultures regardless of the growth temperature. These results thus indicate that the inhibitors would probably be effective at temperatures likely to be encountered under practical conditions.

Mechanistically, the antimicrobial activity of the nitrocompounds has not been resolved, although it has been reported that certain variants of the short-chain nitrocompounds inhibit hydrogen and formic acid metabolism by mixed populations of rumen microbes [30]. This finding indicates that these nitrocompounds may affect hydrogenase or formate hydrogen lyase/formate dehydrogenase activity [30]. Moreover, Angermaier and Simon [43] reported that 2-nitroethanol nonspecifically inhibited ferredoxin-linked hydrogenase activity of *Clostridium pasteurianum*, which may be involved in electron transfer reactions involving hydrogen uptake or evolution in anaerobic ecosystems and in the inhibition of uric acid catabolism [44,45]. Whether or not similar mechanisms may be operative in the *Yersinia* has yet to be determined; however, *Y. ruckeri* is known to possesses a single [NiFe]-containing hydrogenase system [22], and *Y. aleksiciae* contains genes (*hyd*2 and *hyd*4) for two separate [NiFe]-containing hydrogenases [20]. In some cases, similar *Hyd*2 and *Hyd*4 class hydrogenases have contemporarily been considered, depending on the environmental conditions, to be functionally involved in either the evolution or oxidation of hydrogen, the latter supporting the generation of a proton gradient for energy conservation or the reduction of oxidized ferredoxin as an electron carrier [46]. While historically considered exclusively to be anaerobic enzymes, Barz et al. [46] report that at least some hydrogenases may operate in either aerobic or anaerobic conditions. Moreover, microbial hydrogenase activity is recognized as an important energy-conserving enzyme in gastrointestinal environments, but also within aquatic and terrestrial environments, and thus may ultimately be found to be relevant for the survival of *Yersinia* in these environments [46,47,48].

While *Staphylococcus* and *Listeria* lack hydrogenase activity, each express functionally important regulatory proteins containing ferredoxin-like moieties that could possibly explain the inhibitory activity of nitrocompounds against the pathogens reported earlier [15,27]. For instance, *Staphylococcus aureus* produces a signal transduction protein (PstA) that possess a ferredoxin-like domain contributing to its function as a receptor to cyclic diadenosine monophosphate (C-di-AMP), thus affecting the latter protein’s role as an important secondary messenger [49]. Similarly, nitrocompound-caused inhibition of the ferredoxin-like domain in a chlorite-dismutase-like protein (LmCld) produced by *L. monocytogenes* may disrupt its function as a regulator of heme synthesis [50].

Consistent with previous observations for *E. coli*, *Salmonella* and *Campylobacter* [13,28], nitroethane appears, in the present study, to be less potent as an antimicrobial against either *Y. ruckeri* or *Y. aleksiciae* than the nitro-alcohols, which appeared to be near equally effective. Conversely, 2-nitropropanol was found to be more effective than either 2-nitroethanol or nitroethane when tested against the Gram-positive *Staphylococcus* and *Listeria* [15,27]. Moreover, in incubations of mixed populations of ruminal microbes, nitroethane and 2-nitroethanol were more effective at reducing methanogenesis than 2-nitropropanol [29]. It seems reasonable to suspect that the potencies of these nitrocompounds may be impacted by differences in cell wall structure, as the nitro-alcohols appear more effective against Gram-negative bacteria than nitroethane or the substituted nitroalkanes. However, considering the current lack of information regarding the antimicrobial effects of these nitrocompounds, this cell wall hypothesis is made cautiously with the recognition that the possible involvement of other mechanistic factors, such as, but not limited to, those described earlier, cannot be ruled out.

Ethyl nitroacetate and ethyl-2-nitropropionate exhibited activities more similar to nitroethane than to the nitro-alcohols (Table 3), which suggests that the alcohol functional group contributes to the antimicrobial activity of the nitro-alcohols. However, when tested at 9 mM, the nitro-alcohols exhibited greater potency than ethanol and isopropyl alcohol, which are commonly used as disinfectants in medical settings, although usually at 60 to 90% vol/vol [51], rather than the lower concentrations tested here. At 9 mM, ethanol or isopropyl alcohol would be equivalent to 0.1 or 0.14 % vol/vol, respectively. Methanol at 9 mM, the equivalent to 0.07% vol/vol, exhibited no anti-Yersinia activity.

The results from the present study provide new information regarding the differential antimicrobial activity of chlorate and the antimicrobial activity of short-chain nitrocompounds against *Y. ruckeri* and *Y. aleksiciae*. From a practical perspective, the potential development of these antimicrobials into efficacious treatments for controlling enteric redmouth disease will undoubtedly require further study to examine their efficacy, safety and optimum application methods. Considering that *Y. ruckeri* infection occurs mainly via entry of the pathogen through the host’s gill tissues, it seems reasonable to suspect that some type of emersion application method may be needed; however, this needs to be investigated. If the nitrocompounds can be administered in low enough concentrations, it may be possible to add to containment pools, and if catabolized rapidly enough to innocuous products, it may be possible to add the nitrocompounds to small farming ponds. Of course, any administration procedure will likely need to be approved by the appropriate regulatory agencies. Information on nitrocompound toxicity is limited, however, and is not readily extrapolatable between compounds. For instance, while most of the known nitrocompounds appear to be generally innocuous, the naturally occurring nitrocompounds 3-nitro-1-propionate and 3-nitro-1-propanol are toxic if consumed by naive animals [52]. With respect to genotoxicity, this appears to be limited to secondary nitroalkanes, and has not been reported for secondary nitroalcohols or nitroacids or for primary or tertiary nitrocompounds [52,53,54]. Clearly, more research is needed to learn how these compounds negatively impact pathogen survivability, and whether they may ultimately serve useful in preventing enteric redmouth disease.

## Figures and Tables

**Table 1:** Main effects of nitrate, chlorate, 2-nitropropanol and their indicated combinations on mean specific growth rates and maximum optical density during 24 h anaerobic culture of *Yersinia ruckeri* and *Yersinia aleksiciae* in half-strength Brain Heart Infusion broth at 28 °C and 37 °C, respectively.

	Mean Specific Growth Rate (h^−1^)		Maximum Optical Density (OD 600 nm)	
Treatment	*Yersinia ruckeri*	*Yersinia aleksiciae*	*Yersinia ruckeri*	*Yersinia aleksiciae*
None	0.179 ^a^	0.463 ^a^	0.200 ^b^	0.290 ^a^
9 mM Nitrate	0.160 ^a^	0.459 ^a^	0.215 ^ab^	0.281 ^ab^
9 mM Chlorate	0.173 ^a^	0.369 ^b^	0.229 ^a^	0.217 ^c^
9 mM 2-Nitropropanol	<0.001 ^b^	0.014 ^c^	0.045 ^c^	0.050 ^d^
9 mM Nitrate plus 9 mM chlorate	0.170 ^a^	0.427 ^a^	0.228 ^a^	0.278 ^b^
9 mM 2-Nitropropanol plus 9 mM chlorate	<0.001 ^b^	<0.001 ^c^	0.047 ^c^	0.050 ^d^
*p* value	<0.0001	<0.0001	<0.0001	<0.0001
Standard error of the mean	0.0157	0.0107	0.0060	0.0036

^a,b,c,d^ Means (*n* = 3) within columns with unlike superscripts differ at *p* < 0.05.

**Table 2:** Comparison (BLAST*p*) of the putative respiratory nitrate reductase genes from *Yersinia aleksiciae*.

*Y. aleksiciae* Protein ^a^	Percent Amino Acid Identity		
	*Yersinia mollaretii* ^b^	*Escherichia coli str. K-12 substr. MG1655* ^b^	*Yersinia ruckeri* ^b^
Respiratory nitrate reductase alpha chain	98.16	85.98	No significant similarity found
Respiratory nitrate reductase beta chain	97.86	87.97	No significant similarity found
Respiratory nitrate reductase delta chain	92.09	64.90	No significant similarity found
Respiratory nitrate reductase gamma chain	98.68	84.98	No significant similarity found

^a^ Annotated sequences for alpha, beta, delta and gamma chains are available as Supplemental Material. ^b^ *Y. mollaretii*, GenBank GCA_001108385.1, closest match; *E. coli str. K-12 substr. MG1655*, Genbank GCA_000005845.2; *Y. ruckeri*, GenBank: GCA_017498685.1.

**Table 3:** Comparative antimicrobial effects of select nitrocompounds and commonly used alcohols during 12 h anaerobic culture of *Yersinia ruckeri* and *Yersinia aleksiciae* in half-strength Brain Heart Infusion broth at 28 °C and 37 °C, respectively.

Treatment	Mean Specific Growth Rate (h^−1^)		Maximum Optical Density (OD 600 nm)	
	*Yersinia ruckeri*	*Yersinia Aleksiciae*	*Yersinia ruckeri*	*Yersinia aleksiciae*
None	0.424 ^a^	0.364 ^ab^	0.233 ^a^	0.176 ^a^
9 mM 2-Nitropropanol	<0.001 ^d^	<0.001 ^d^	0.005 ^e^	<0.001 ^d^
9 mM 3-Nitropropanol	<0.001 ^d^	<0.001 ^d^	0.004 ^e^	<0.000 ^d^
9 mM 2-Nitroethanol	<0.001 ^d^	<0.001 ^d^	0.005 ^e^	0.002 ^d^
9 mM Nitroethane	0.082 ^c^	0.141 ^c^	0.047 ^b^	0.007 ^d^
9 mM Ethyl nitroacetate	0.165 ^b^	0.146 ^bc^	0.076 ^d^	0.081 ^c^
9 mM Ethyl-2-nitropropionate	0.237 ^b^	0.144 ^c^	0.115 ^b^	0.074 ^c^
9 mM Ethanol	0.401 ^a^	0.387 ^a^	0.213 ^a^	0.172 ^a^
9 mM Methanol	0.456 ^a^	0.344 ^abc^	0.226 ^a^	0.186 ^a^
9 mM Isopropanol	0.460 ^a^	0.327 ^abc^	0.222 ^a^	0.132 ^b^
*p* value	<0.0001	<0.0001	<0.0001	<0.0001
Standard error of the mean	0.0262	0.0904	0.0083	0.0094

^a,b,c,d,e^ Means (*n* = 3) within columns with unlike superscripts differ at *p* < 0.05.

**Table 4:** Comparative effects of levels of 2-nitropropanol on mean specific growth rates and maximum optical densities during 12 h anaerobic culture of *Yersinia ruckeri* and *Yersinia aleksiciae* in half-strength Brain Heart Infusion broth at 28 °C and 37 °C, respectively.

Treatment	Mean Specific Growth Rate (h^−1^)		Maximum Optical Density (OD 600 nm)	
	*Yersinia ruckeri*	*Yersinia aleksiciae*	*Yersinia ruckeri*	*Yersinia aleksiciae*
None	0.230 ^a^	0.327 ^a^	0.255 ^a^	0.187 ^a^
2.25 mM 2-Nitropropanol	0.012 ^b^	<0.001 ^b^	0.022 ^c^	0.027 ^b^
4.5 mM 2-Nitropropanol	0.008 ^b^	<0.001 ^b^	0.020 ^c^	0.013 ^b^
9 mM 2-Nitropropanol	<0.001 ^b^	<0.001 ^b^	0.022 ^c^	0.014 ^b^
*p* value	<0.0001	0.0077	<0.0001	<0.0001
Standard error of the mean	0.0182	0.0775	0.0038	0.0032

^a,b,c^ Means (*n* = 3) within columns with unlike superscripts differ at *p* < 0.05.

**Table 5:** Main effects of nitrocompound and chlorate treatment on log-fold reductions of *Yersinia ruckeri* and *Yersinia aleksiciae* after 24 h culture with mixed populations of porcine gut or tilapia tank water microbes.

Treatment	Net Change in log_10_ Colony Forming Units/mL Incubation Mixture	
	*Yersinia ruckeri* Cultured Aerobically at 28 °C in Freshly Collected Tilapia Fish Water	*Yersinia aleksiciae* Cultured Anaerobically at 37 °C in Half-Strength BHI Broth Inoculated with 0.2% wt/vol Freshly Collected Pig Feces
None	0.94 ^a^	−0.33 ^a^
9 mM 2-Nitropropanol	−4.63 ^e^	−4.83 ^d^
9 mM 2-Nitroethanol	−4.63 ^e^	−4.83 ^d^
9 mM Nitroethane	−0.14 ^d^	−1.18 ^c^
9 mM Ethyl nitroacetate	0.81 ^b^	−0.82 ^b^
9 mM Ethyl-2-nitropropionate	0.40 ^c^	−0.83 ^b^
9 mM Sodium chlorate	1.38 ^a^	−1.25 ^c^
*p* value	<0.0001	<0.0001
Standard error of the mean	0.1031	0.6400

^a,b,c,d,e^ Means (*n* = 3) within columns with unlike superscripts differ at *p* < 0.05. Initial concentration of *Yersinia ruckeri* and *Yersinia aleksiciae* at 0 time in their respective incubation mixtures were 4.93 ± 0.04 and 5.13 ± 0.06 log_10_ CFU/mL.

## Data Availability

Whole genome sequences for *Yersinia mollaretii, Escherichia coli str. K-12 substr. MG1655*, *Yersinia ruckeri* and *Yersinia aleksiciae* are GenBank number GCA_001108385.1, Genbank number GCA_000005845.2; GenBank number GCA_017498685.1 and Genbank number PRJNA854595. Annotated sequences for alpha, beta, delta and gamma chains are available as Supplemental Material.

## Supplementary Materials

The following are available online at https://www.mdpi.com/article/10.3390/pathogens11111381/s1, Table S1: Yersinia alekisciae Annotations.
